# Compositional Effects on Indentation Mechanical Properties of Chemically Strengthened TiO_2_-Doped Soda Lime Silicate Glasses

**DOI:** 10.3390/ma15020577

**Published:** 2022-01-13

**Authors:** Stefan Karlsson

**Affiliations:** Glass Unit, Department of Building and Real Estate, Division of Built Environment, RISE Research Institutes of Sweden, Vejdes plats 3, 352 52 Växjö, Sweden; stefan.karlsson@ri.se

**Keywords:** glass, chemical strengthening, ion exchange, nano-indentation, micro-indentation, mechanical properties

## Abstract

TiO_2_ is an important oxide for property modifications in the conventional soda lime silicate glass family. It offers interesting optical and mechanical properties, for instance, by substituting heavy metals such as lead in consumer glasses. The compositional effects on the hardness, reduced elastic modulus and crack resistance as determined by indentation of chemically strengthened (CS) TiO_2_-doped soda lime silicate glass was studied in the current paper. The CS, which was performed by a K^+^ for Na^+^ ion exchange in a molten KNO_3_ salt bath at 450 °C for 15 h, yielded significant changes in the indentation mechanical properties. The hardness of the glass samples increased, and this was notably dependent on the SiO_2_, CaO and TiO_2_ content. The reduced elastic modulus was less affected by the CS but showed decrease for most samples. The crack resistance, an important property in many applications where glasses are subjected to contact damage, showed very different behaviors among the series. Only one of the series did significantly improve the crack resistance where low CaO content, high TiO_2_ content, high molar volume and increased elastic deformation favored an increased crack resistance.

## 1. Introduction

Soda lime silicate (SLS) glass compositions dominate a wide range of industrial applications [1]. For instance, SLS glass is used in windows, containers, household glasses, displays, cover glasses and in automotive glazing. The industrial importance of this glass composition originates from its forming ability that yields a low-cost manufacturing process [2] but also transparency in the visible range, relatively high hardness and good chemical durability. However, due to their brittle, fracturing nature and their low resistance to surface defects, the practical strength of commercially available glass products is low [3]. As a route toward sustainable development [4], reducing the thickness of glass products is important as it affects sustainability in multiple ways, e.g., by resource efficiency, energy efficiency and fuel efficiency. The routes towards thinner and stronger glass are multiple [3], of which the strengthening of glass [5] is one route besides damage-resistant glass [6] and defect-free glass production [7]. Strengthened glass products are also used in a wide variety of applications, e.g., architectural, automotive, containers, displays, cover glasses and household glasses. Thermal strengthening is dominating but is less suitable for thin glass (i.e., below 2 mm) whereas chemical strengthening (hereinafter called CS) has reached wide market success, especially for electronic handheld devices [5], pharmaceutical auto-injectors [8], aircraft windshields [8] and high-speed trains [9]. However, CS has also found applications in other market segments, e.g., photovoltaics [10,11,12], automotive [13], wine glasses (e.g., the Stem Zero collection from Nude Glass), architectural applications [14] and flexible photonics [15].

CS is based on an ion exchange of smaller ions in the glass with larger ions from a salt bath, e.g., typically Na^+^ for K^+^ or Li^+^ for Na^+^ ion exchange. There is also the possibility of performing a two-step ion exchange (e.g., Li^+^ for Na^+^ and Na^+^ for K^+^ ion exchange) and thereby tailoring the stress profile, the so-called Engineered Stress Profile (ESP). The requirements for a well-performing CS glass are a high exchange rate of ions, high build-up of compressive stresses, low stress relaxation and high damage resistance [5,16,17]. Aluminosilicate and boroaluminosilicate glasses are typically high-performance glass compositions [18]; however, they are compositions with a set of properties that are not suited for all possible applications, e.g., for optical applications. The alumina content, which should ideally be in the range of the alkali content, also implies a high viscosity and high melting temperature [19,20,21]. It is therefore interesting to investigate the CS of other glass compositions in terms of kinetics, build-up of compressive stresses, stress relaxation and damage resistance.

Indentation is a powerful tool for investigating mechanical properties through which it is possible to determine the hardness, elastic modulus, and crack-initiation probability [22]. The mechanical properties of glasses have recently received much interest and they are of great importance when designing glasses for specific products [3]. Cover glass for electronic handheld devices is, for instance, subjected to different sources of mechanical contact damage and thus the mechanical properties are of great importance [23]. Other products in very different applications, e.g., wine glasses, are also subjected to different sources of contact damage, for instance, during handling and dishwashing. The latter also give demands on the chemical durability. In both product examples, cover glass and wine glasses, damage resistance is beneficial for the product and both examples are topics that most people can relate to.

TiO_2_ is an interesting component for incorporation into silicate glass mainly owing to its optical properties [24,25,26,27,28,29,30,31]. It has been used as a coloring agent [32,33], UV-protective agent [34,35,36] and for tailoring optical properties for different applications, e.g., optical glasses and lead-free crystal glass [24,37]. It has also found uses as a nucleating agent [38] for glass ceramics. TiO_2_ in glass has recently been studied more frequently for its effect on the structural, thermal and mechanical properties [39,40,41,42,43]. TiO_2_ is classified as an intermediate network former which means that it can act as both a network former and as a network modifier; however, in the soda-(lime)-silicate-glass system it mainly acts as a network former. TiO_2_, as it replaces SiO_2_, affects the mechanical properties by increasing the hardness and elastic modulus [39,41]. The crack initiation resistance was found to depend on Poisson’s ratio and a minimum was found around ν = 0.21. As SiO_2_ is replaced by TiO_2_, Poisson’s ratio increases. The effect of TiO_2_ on CS and the resulting properties has previously not been reported in the scientific literature; thus, the current paper will provide a novel study on the effect of CS on the mechanical properties in TiO_2_-containing soda lime silicate glass compositions. In a related paper, the effect of ion-exchange kinetics, structure and optical properties of some selected soda lime titanosilicate glasses was studied [44].

## 2. Materials and Methods

### 2.1. Glass Compositions

The compositional variations of the glass samples originate from a conventional SLS composition, and then the compositional changes categorize them into three different series while still keeping the Na_2_O relatively constant, as described in detail in [24] and given in Table 1. In summary, in Series 1 the SiO_2_ and TiO_2_ increases while the CaO decreases, in Series 2 the TiO_2_ replaces SiO_2_ and in Series 3 the TiO_2_ is replacing CaO. The glasses were molten in Pt/Rh10 crucibles at 1450 °C for 18 h followed by 1 h of homogenization by stirring with a Pt/Rh10 flag and a conditioning step at 1500 °C for 2 h before pour quenching into non-tempered steel molds. Silica sand (MAM1S, Sibelco, Antwerp, Belgium) and reagent grade NaNO_3_ (Scharlab, Barcelona, Spain, ≥99.5%), Na_2_CO_3_ (anhydrous, Fisher Scientific, Waltham, MA, USA, ≥99.5%), CaCO_3_ (Sigma Aldrich, St. Louis, MO, USA, ≥99.0%) and TiO_2_ (Acros Organics, Geel, Belgium ≥98.0) were used as raw materials. Annealing was performed at 550–580 °C for 1 h before conventional slow cooling of 0.5 °C/min to 400–430 °C and further cooling to ambient temperature by 2 °C/min. The chemical compositions that were analyzed using Laser Ablation Inductively Coupled Plasma Mass Spectrometry (LA-ICP-MS) (see [24,39] for details), are given in Table 1. The glass compositions will hereinafter be referred to as Series 1, Series 2 and Series 3 and the samples 1.1, 1.2, etc., where the first number denotes the series number and the second the sample in that series. However, please note that sample 1.1 is the start composition of each series, such that 1.1 = 2.1 = 3.1.

The density (*ρ*) was measured using Archimedes’ principle in distilled water and the values of the glass samples were calculated using the expression

(1) $$\rho =\frac{m_{d}}{m_{d}-m_{w}}\cdot \rho_{w}$$

where *m_d_* is the weight of the dry glass sample, *m_w_* is the weight of the sample immersed in deionized water and *ρ_w_* is the density for water. Sample weights varied between 6 and 10 g and the densities were estimated to be reproducible within the uncertainty of ±0.2%.

The molar volume of the glass sample was obtained by using the conventional expression

(2) $$V_{m}=\frac{\sum_{i}x_{i}M_{i}}{\rho }$$

where *x_i_* is the molar fraction, *M_i_* is the molar mass of element *i* and *ρ* is the density of the glass. The atomic packing density was calculated according to

(3) $$C_{g}=\frac{\sum_{i}x_{i}V_{i}}{V_{m}}$$

where *V_i_* is the volume for oxide *i* (*i = M_x_O_y_* where *M* is the cation and *O* the oxygen) calculated by $V_{i}=\frac{4}{3}\pi N\left(xr_{M}^{3}+yr_{O}^{3}\right)$, *N* is Avogadro’s constant and *r* is the ionic radii of the corresponding cations and oxygen [45]. The ionic radii data were taken from [46] with the following coordination numbers (*CN_i_*) for the ions *i*; Si^4+^: *CN_Si_* = 4 and *r_Si_* = 0.26 Å, O^−2^: *CN_O_* = 2 and *r_O_* = 1.35 Å, Na^+^: *CN_Na_* = 8 [47] and *r_Na_* = 1.18 Å, Ca^2+^: *CN_Ca_* = 8 [47] and *r_Ca_* = 1.12 Å as well as Ti^4+^: *CN_Ti_* = 4 [39] and *r_Ti_* = 0.42 Å. The oxygen packing density (*C_Ox_*) was calculated according to

(4) $$C_{Ox}=\frac{1}{V_{m}}\underset{i}{\sum }x_{i}N_{ox,i}$$

which is the molar amount of oxygen per unit volume of glass [48,49]. *N_ox,i_* is the stoichiometric number of oxygen atoms provided by each glass component, i.e., *N_ox,Si_* = 2, *N_ox,Na_* = 1, *N_ox,Ca_* = 1 and *N_ox,Ti_* = 2.

### 2.2. Ion-Exchange Procedure

Pre-polished glass pieces were subjected to a thermally assisted ion-exchange process in a Hybe muffle furnace (model KUT180) at 450 °C. The glass pieces were put into a metal mesh basket and were initially heated next to a stainless-steel container that contained KNO_3_ salt (reagent grade from Acros Organics, Geel, Belgium ≥99%). The salt was first melted at 450 °C before the metal mesh basket with the glass pieces was put inside the salt bath for 15 h. Then the metal mesh basket with the glass samples inside was removed from the salt bath and placed next to the steel container inside the furnace. The furnace was turned off and the door was left open by a couple of centimeters so that the cooling was about 3–5 °C/min. The salt bath temperature was controlled to be within ±5 °C of the target temperature using a P655 logger from Dustmann Electronic GmbH and a thermocouple of type K. The samples and series that were subjected to the ion-exchange strengthening are referred to as chemically strengthened (CS), e.g., Series 1-CS.

### 2.3. Indentation Characterizations

The hardness (*H*) and reduced elastic modulus (*E_r_*) was measured by nano-indentation using Oliver and Pharr’s method [50] on polished glass samples before and after ion-exchange treatment. Hardness is defined by *H* = *F_m_*/*A_p_*, where *F_m_* is the maximum applied load and *A_p_* is the projected contact area. *A_p_* is calculated by a fitting polynomial, $A_{p}\left(h_{c}\right)=C_{0}h_{c}^{2}+C_{1}h_{c}^{1}+C_{2}h_{c}^{1/2}+C_{3}h_{c}^{1/4}+\ldots +C_{8}h_{c}^{1/128}$ where *C_x_* are indenter tip specific factors (*C*_0_ = 24.56 for a perfect Berkovich indenter) and *h_c_* is the real contact depth considering the sink-in effect, calculated from Oliver and Pharr’s method, $h_{c}=h_{m}-\varepsilon \frac{F_{m}}{S}$, where *h_m_* is the initial contact depth, *ε* is a tip factor (*ε* = 0.72 for a Berkovich tip) and *S* is the stiffness as determined from the slope during unloading, $S=\frac{\partial P}{\partial h}$. The reduced elastic modulus is determined through $E_{r}=\frac{\sqrt{\pi }}{2\beta }\frac{S}{\sqrt{A_{p}}}$, where *β* is a geometrical tip factor (*β* = 1.034 for a Berkovich indenter).

The nano-indenter was an Anton Paar NHT^2^ instrument. The measurements were made with the loads 1, 5, 10, 15, 25, 50 and 75 mN. For the 1 mN load, 40 indents were made (except for sample 3.5 where only 20 indents at 1 mN were measured) while 20 indents were made for each of the other load levels. In some cases, one indentation outlier datapoint, or for the 1 mN load, sometimes two datapoints, was removed from the analyzed data due to unrealistic scattering. The full information on the amount of data is given in the dataset.

The acquisition rate for the nano-indentation was 10 Hz, the loading-and-unloading rate was twice the load per minute (e.g., 2 mN/min for max load of 1 mN), the holding time at the max load was 10 s, the approach distance was 3 µm, the approach speed was 4 µm/s, the retract speed was 6 µm/s, the frame compliance was 0.2 µm/N and the stiffness threshold was 500 µN/µm. The Poisson’s ratio (*ν*) values were taken from [39]. The Berkovich tip geometry was calibrated using a certified standard sample made of fused quartz.

Crack resistance was measured by micro-indentation on the polished and ion-exchange-treated glasses using a Micro-Combi Tester (MCT) from CSM Instruments. Fifteen indents for each load were made using a diamond Vickers indenter. The micro-indentations were made with an acquisition rate of 10 Hz using linear loading with a load-and-unload rate of two times the load per minute, and the holding time at the load was 15 s. The approach speed was 8 µm/min and the retract speed was 16.6 µm/min, the contact force was 25 mN and the contact stiffness threshold was 25,000 µN/µm. The crack-resistance method is described in detail in [51] and follows the original procedure of Kato et al. [52] and Wada et al. [53]. The least-square method was used for the fitting procedure using the Weibull sigmoidal function,

(5) $$PCI=1-\exp^{-{\left(x/x_{c}\right)}^{m}}$$

where *PCI* is the probability of radial crack initiation, *x* is the load, *x_c_* is the characteristic value and *m* is the Weibull modulus. The crack resistance is then defined as the load when the *PCI* is 50%. However, using the Weibull cumulative function, *x_c_* can be used as a relative measure of the crack resistance. The characteristic value, *x_c_*, has the property that when *x* = *x_c_*, the *PCI* is equal to 1 − e^−1^, which is approximately 0.632 or 63.2%. All indentations, both nano and micro, were performed in an environment with a temperature of 23 ± 2 °C and a relative humidity of 40 ± 10%.

## 3. Results

### 3.1. Hardness and Reduced Elastic Modulus from Nano-Indentation

**Figure 1:**
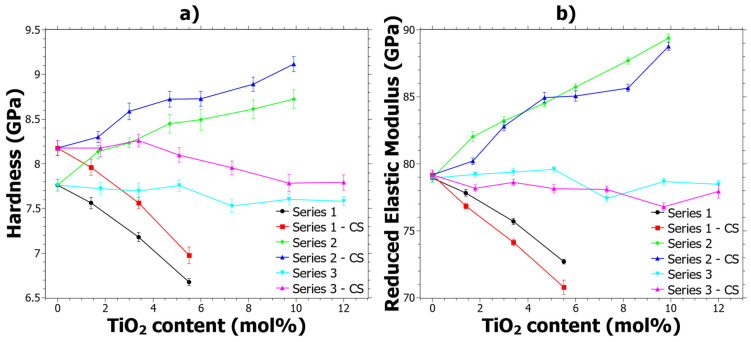
Properties determined by 75 mN nano-indentation before and after ion exchange: (**a**) Hardness and (**b**) Reduced Elastic Modulus (*E_r_*). The error bars are given from the standard deviations of the measurements. Series 1 refers to samples 1.1 to 1.4, Series 2 to samples 2.2 to 2.7, Series 3 to 3.2 to 3.7 and CS refers to values after ion-exchange strengthening.

The implications of CS on the mechanical properties are primarily influenced by the compressive stresses that arise due to the exchange of larger ions into the glass from the molten salt bath. In fact, the mixed alkali effect [54] is generally not seen in CS glasses [55] and therefore the hardness generally increases with CS. In Figure 1a it can be seen that the hardness as a function of TiO_2_ content follows trends similar to what has previously been reported [39], and that the CS glasses generally exhibit a shift from the original reference hardness. The increase in hardness is generally about 5 ± 2%, but for some samples with higher TiO_2_ content it is slightly lower, around 2 ± 1%, while the errors for all samples are quite similar. Hereinafter, the difference in hardness will be called Δ*H_CS_*, which is given by Δ*H_CS_* = *H_CS_* − *H_ref_*. However, any explicit trends regarding the Δ*H_CS_* are not seen from the data in Figure 1a; instead, the trends are discussed in the next section where hardness as a function of load is discussed.

The reduced elastic modulus (*E_r_*) shown in Figure 1b shows different trends compared to the hardness. In general, most of the samples show a decrease of *E_r_* because of the CS. The reduction of *E_r_* is in contrast to aluminosilicate glass, in which an increase is observed for alumina contents larger than 4 mol% [55]. However, it is important to note that the *E_r_* is dependent on Poisson’s ratio, which was assumed not to change with the CS.

### 3.2. Hardness as a Function of Indentation Load from Nano-Indentation

**Figure 2:**
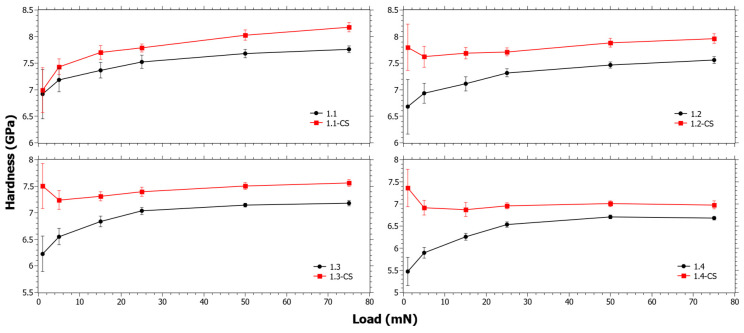
Nano-indentation hardness as a function load for Series 1 before and after chemical strengthening (CS).

In Figure 2, the hardness before and after CS for Series 1 can be seen. At low loads Δ*H_CS_* is increasing with TiO_2_ content. It is suspected that the indentation at low loads is affected by the stress relaxation that occurs during CS. In a previous study [56], it was observed that the SiO_2_ content had close to a linear correlation to the viscosity in these compositions. Thus, as SiO_2_ is increases, the viscous relaxation is reduced. However, the stress relaxation is also dependent on the inherent instantaneous stress relaxation that occurs in CS [57]. Either way, the trend of the Δ*H_CS_* is likely strongly influenced by the increasing SiO_2_ content (Table 1). The trends in Figure 2 are also affected by the ion-exchange kinetics, and since Δ*H_CS_* is clearly decreasing with increasing load it is reasonable that the ion-exchange interdiffusion coefficient is decreasing in that series. In Figure S1 in the Supplementary Materials, the Δ*H_CS_* for Series 1 as a function of different components is shown and it also reveals that as a function of TiO_2_, Δ*H_CS_* has a similar trend to SiO_2_, but the opposite of CaO.

**Figure 3:**
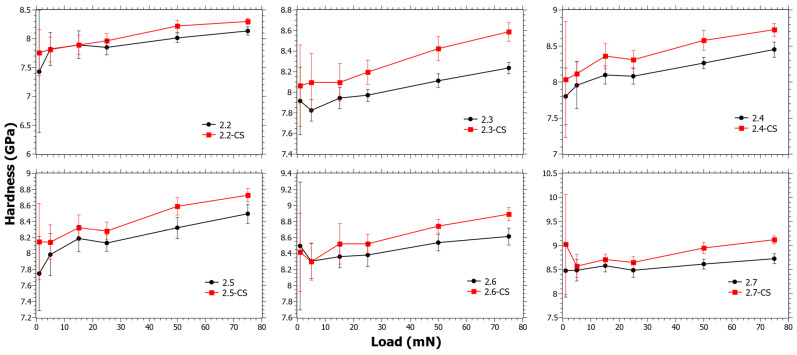
Nano-indentation hardness as a function load for Series 2 before and after chemical strengthening (CS).

In Figure 3, the trend changes resulting from the CS are small and the values of Δ*H_CS_* are within error or slightly above. In Figure S2 in the Supplementary Materials, it is also shown that Δ*H_CS_* does not reveal any trend as a function of the SiO_2_, TiO_2_ or CaO content. Regardless, from studying the data closely it can be seen that the trends at low loads are different from those at high loads, but it must be considered that the error at low loads is also much higher. It is known from a separate study that the ion-exchange interdiffusion coefficient decreases with increasing TiO_2_ content [44]. However, as *E_r_* increases with the TiO_2_ content, a hypothesis is that the instantaneous stress relaxation becomes lower with increasing TiO_2_ content, but at the same time the viscous relaxation increases due to the suppressing effect of TiO_2_ on viscosity [56]. This can explain why the trends at low loads are slightly different from those at high loads.

**Figure 4:**
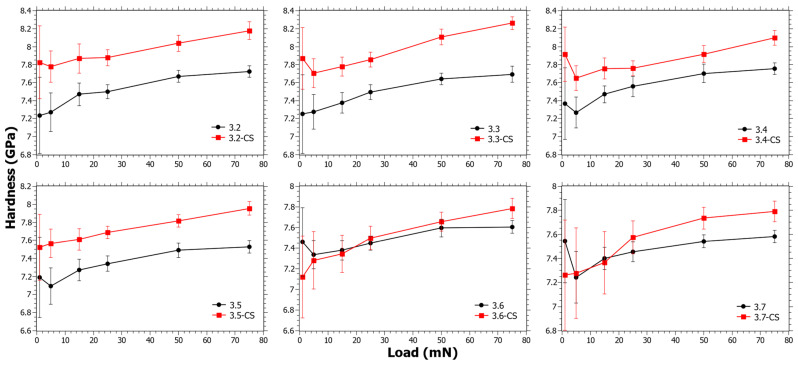
Nano-indentation hardness as a function load for Series 3 before and after chemical strengthening (CS).

In Figure 4, the hardness as a function of load for Series 3 can be seen. The Δ*H_CS_* generally has a decreasing trend with increasing TiO_2_ content (see Figure S3 in the Supplementary Materials). In Series 3, the SiO_2_ content is almost the same (Table 1) and in principle only the CaO is reduced. TiO_2_ is nevertheless considered likely to reduce the ion-exchange kinetics, but at the same time the reduction of Ca^2+^ increases the ionic mobility. It is well known that Ca^2+^ hinders ionic mobility, not only in the application of ion exchange [55,58] but also in the application of ionic conductivity [59]. For samples 3.6 and 3.7 there is a trend change as Δ*H_CS_* is negative at low loads. However, whether this depends on the low CaO content, the coordination change for Ti^4+^ [39], or a combination of the two is impossible to say.

In summary, since the three series are very different in terms of their composition (Table 1) and nature, which is also reflected in the trends of their properties (Figure 1), there is not a simple explanation for the trend in Δ*H_CS_*. As can be seen from Figures S1–S3 in the Supplementary Materials, there is not a single definitive correlation for all three series. Instead, there are likely several causes of the Δ*H_CS_* trend. However, it is possible to draw some general observations based on the results: (1) increasing the SiO_2_ content tends to increase the Δ*H_CS_*; (2) increasing the TiO_2_ content tends to slightly decrease Δ*H_CS_*; and (3) increasing the CaO content tends to give an increasing trend of Δ*H_CS_* with a maximum around 9 mol%. These observed trends are probably primarily dependent on the compressive-stress profiles of the different samples.

### 3.3. Crack Resistance

The crack resistance (CR) is an important property of CS glass as it helps to determine the service lifetime of the glass. An increasing CR generally reduces the probability of inflicted critical flaws and ultimately determines the strength of the glass. As an indenter is pressed into a glass, the energy is dissipated either as elastic or plastic deformation; however, if the glass’s ability to deform is not sufficient then it will crack [22,60]. Most frequently, glass shows radial and half-penny cracking and depending on the method of CR, the probability of crack initiation can be measured [52]. The understanding of CR is of high interest. There are many studies trying to elucidate the underlying parameters affecting crack resistance [6,22,52,60,61,62,63,64,65,66,67,68,69,70,71,72]. Their purpose is to advance the understanding of glass science and provide the possibility of modeling CR with the aim of developing more crack-initiation-resistant glasses [3,73,74].

**Figure 5:**
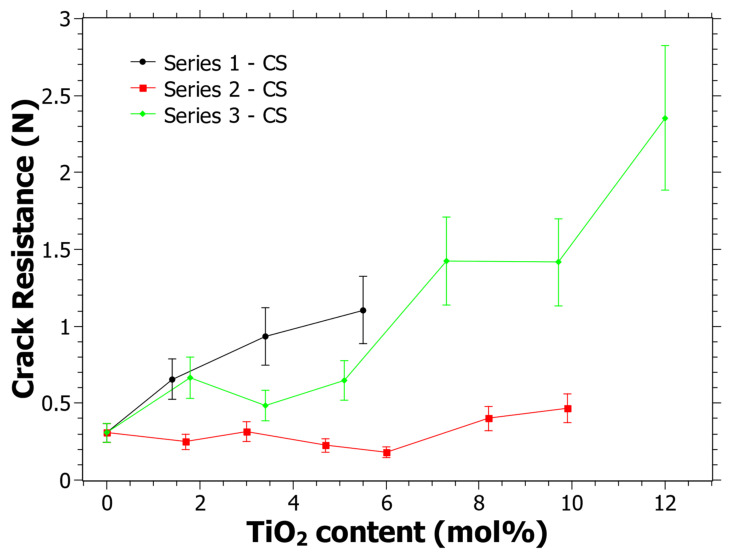
Crack resistance (CR) as determined using a Vickers indenter and when the percentage of radial crack initiations is equal to 50% following the procedure in [51]. Estimated error is 20% of the CR value and is shown by the error bars in the graph.

In Figure 5, the CR as a function of the TiO_2_ content can be seen. The three different series show different behavior. It is clear from Figure 5 that Series 1 and Series 3 have an increasing trend in the CR as the TiO_2_ content increases. R^2^ is 0.95 and 0.88 for Series 1 and Series 3, respectively, while the R^2^ for Series 2 is 0.42. Linear fitting gives positive derivatives for all functions, including Series 2. The trend for all series is thus similar in the sense that the CR tends to increase with the TiO_2_ content. Although, it is noted that the CR is lower than previously reported CR values that were not subjected to CS, at least for Series 1 and 2 [39]. Note, since neither the same indenter instrument, the same indenter tip nor the same loads were used, the results cannot be directly compared to the previous study. However, it has previously been observed that the CR may become lower with CS [55,75] and it is quite likely that this is the case for some of the studied compositions here as well.

**Figure 6:**
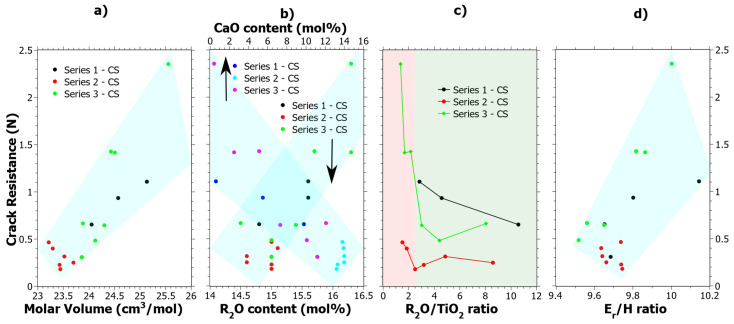
Crack resistance correlation to (**a**) Molar Volume (*V_m_*), (**b**) R_2_O content where R = Na or K, (**c**) Ti^4+^ coordination as shown by the R_2_O/TiO_2_ ratio where the red semi-transparent color indicates a mixture of four- and five-coordinated Ti^4+^ and the green color primarily tetrahedrally coordinated Ti^4+^ and (**d**) the *E_r_/H* ratio as calculated from the data in Figure 1, i.e., 75 mN nano-indentation load. The semi-transparent colored areas are guides for the eye.

Series 3 after CS stands out in terms of CR values, which show a clear increase as compared to the values in the unstrengthened state [39]. The CR results given in Table S1 were cross correlated to all the data given in Table 1, and four general observations could be made, which are shown in Figure 6. In Figure 6a, it is shown that increasing the molar volume generally has a positive effect on the CR. In Figure 6b, the compositional effects are shown, whereby increasing R_2_O (where R = Na or K) and decreasing CaO content tend to increase the CR. Both Figure 6a,b show quite clear correlations. In Figure 6c, the CR as a function of Na_2_O/TiO_2_ ratio is shown. Previous studies of the structural effects in these glass samples explained that a coordination change occurs when the Na_2_O/TiO_2_ ratio is about 2.5 [24,39], which has also been observed by others [76,77]. In Figure 6c, the coordination change is shown by the different background colors where green is the tetrahedral coordination and red is the mix of [TiO_4_] and [TiO_5_] polyhedra. In the previous study [39], it was concluded that Ti^4+^ mostly exists in the tetrahedral configuration up to about 10 mol% of TiO_2_ and upon further increase of the [TiO_5_] polyhedral form. At TiO_2_ content levels exceeding 15 mol% the [TiO_5_] polyhedral is the dominating coordination of Ti^4+^. In Figure 6d, CR is generally seen to increase as a function of the *E_r_/H* ratio, which is calculated from the nano-indentation data of the CS samples in Figure 1a,b. *E_r_/H* is a ratio that has previously been noted to effect crack-initiation behavior in oxide glasses [63] and it is correlated to the elastic volume recovery [78]. Since more of the energy from the indentation can be stored by elastic deformation as the *E_r_/H* is increased, it is reasonable that the CR increases. Radial cracking is a result of the elastic-plastic mismatch in the stress field upon unloading. Hypothetically, by increasing the elastic part, a better balance is achieved which results in an increased CR.

## 4. Discussion

The indentation mechanical properties of glasses are today an important topic in glass science, and it is well known that isostatic compression gives enhanced mechanical properties, such as the CS of glass. CS generally yields an increase in the hardness, but a less apparent change is the reduced elastic modulus, and the underlying reasons for this are probably primarily caused by the CS-induced compressive-stress profiles. Therefore, it is planned to study the stress profiles and the stress relaxation of these glasses in the future in order to understand the mechanical properties as a function of indentation load.

The crack resistance after CS does seem to be somehow connected to the coordination of Ti^4+^. However, from this study it is unclear why, and this needs further investigation. One possibility would be the analogy to Si^4+^, which is mainly in a tetrahedral coordination, but can become five-coordinated under pressure [79]. In molecular-dynamics simulations, it has been shown that five-coordinated Si^4+^ has a higher propensity to carry out local shear deformation than four-coordinated Si^4+^ [80]. Perhaps a similar phenomenon occurs for Ti^4+^ when it is five-coordinated. However, the effect of the Ti^4+^ coordination could be in combination with the elastic volume recovery, as indicated by the general increase as a function of *E_r_/H*.

## 5. Conclusions

In the current study, it was found that the compositional changes affected the mechanical properties after chemical strengthening (CS). The results led to several general observations that will serve as the conclusion of the study. A new term, Δ*H_CS_*, was introduced that denotes the difference in hardness before and after CS. Increasing SiO_2_ and CaO contents tended to increase Δ*H_CS_*, the latter by up to around 9 mol%, while increasing TiO_2_ tended to slightly decrease Δ*H_CS_*. Crack resistance, which is an established method for studying crack initiation, showed that CS possibly decreases the crack resistance value for most samples compared to the non-strengthened glass. However, not for Series 3, where a clear increase was seen. Given the compositional changes, the crack resistance is favored by an increased molar volume, lower CaO content, higher Na_2_O content and high TiO_2_ content where Ti^4+^ is both four and five-coordinated. The crack resistance also generally correlates with the ratio reduced elastic modulus over hardness (E_r_/H), which is related to the elastic volume recovery upon unloading. The causes of the observed trends will be further clarified by studying the induced compressive-stress profiles in a future study.

## Figures and Tables

**Table 1:** Normalized glass compositions in mol% as analyzed by LA-ICP-MS, density (*ρ*), molar volume (*V_m_*), atomic packing density (*C_g_*) and oxygen packing density (*C_ox_*). The data were taken from refs [24,39] apart from *C_ox_*.

Label	SiO_2_	CaO	Na_2_O	TiO_2_	*ρ* (g/cm^3^)	*V_m_* (cm^3^/mol)	*C_g_*	*C_Ox_*
Series 1								
1.1	73.9	11.2	15.0	0.0	2.514	23.86	0.499	0.07292
1.2	73.9	9.8	14.8	1.4	2.503	24.05	0.497	0.07286
1.3	75.6	5.5	15.6	3.4	2.478	24.57	0.494	0.07289
1.4	78.3	0.6	15.6	5.5	2.443	25.13	0.490	0.07315
Series 2								
2.2	69.8	14.0	14.6	1.7	2.560	23.70	0.499	0.07242
2.3	68.3	14.0	14.6	3.0	2.566	23.52	0.502	0.07280
2.4	67.0	13.3	15.0	4.7	2.594	23.43	0.505	0.07329
2.5	65.8	13.2	15.0	6.0	2.605	23.44	0.505	0.07328
2.6	62.8	13.9	15.1	8.2	2.638	23.29	0.507	0.07342
2.7	61.3	13.8	15.0	9.9	2.661	23.21	0.509	0.07376
Series 3								
3.2	71.6	12.1	14.5	1.8	2.519	23.88	0.497	0.07261
3.3	71.5	10.1	15.0	3.4	2.520	24.12	0.495	0.07251
3.4	72.3	7.3	15.4	5.1	2.526	24.30	0.497	0.07306
3.5	71.9	5.1	15.7	7.3	2.525	24.43	0.497	0.07334
3.6	71.6	2.5	16.3	9.7	2.535	24.51	0.500	0.07402
3.7	71.3	0.4	16.3	12.0	2.535	25.55	0.497	0.07174
Uncertainties	±1.3	±0.3	±0.3	±0.2	±0.2%	-	-	-

## Data Availability

The datasets generated for this study can be found in the Swedish National Data Service Repository: https://snd.gu.se/en (accessed on 1 December 2021), doi:10.5878/2rze-dy74.

## Supplementary Materials

The following are available online at https://www.mdpi.com/article/10.3390/ma15020577/s1. Figure S1: Δ*H_CS_* of Series 1 for different nano-indentation loads as a function of (a) SiO_2_, (b) TiO_2_ content and (c) CaO content. Figure S2: Δ*H_CS_* of Series 2 for different nano-indentation loads as a function of (a) SiO_2_, (b) TiO_2_ content and (c) CaO content. Figure S3: Δ*H_CS_* of Series 3 for different nano-indentation loads as a function of (a) SiO_2_, (b) TiO_2_ content and (c) CaO content. Table S1: The crack resistance (CR) results as well as fitting data *x_c_* (characteristic value) and *m* (Weibull modulus) for the Weibull fit.
