# Supplementary material for: Compositional Effects on Indentation Mechanical Properties of Chemically Strengthened TiO2-Doped Soda Lime Silicate Glasses
Source: Materials (Basel). 2022 Jan 13;15(2):577. doi: 10.3390/ma15020577 (PMC8779215; doi:10.3390/ma15020577)
Supplement: Supplementary file 1 [file materials-15-00577-s001.zip › materials-1517905-supplementary.pdf]

materials

MDPI

Supplementary Materials

Compositional Effects on Indentation Mechanical Properties of Chemically Strengthened TiO_2 Doped Soda Lime Silicate Glass

Stefan Karlsson

Glass Unit, Department of Building and Real Estate, Division of Built Environment, RISE Research Institutes of Sweden, Vejdes plats 3, SE-352 52 Växjö, Sweden; stefan.karlsson@ri.se

Figure S1, S2 an S3 show the ΔH_{CS} as a function of SiO_2, TiO_2 and CaO content for series 1, series 2 and series 3 respectively. Please note that no error bars are shown but the error bars from Figures 2, 3 and 4 can work as a guide.

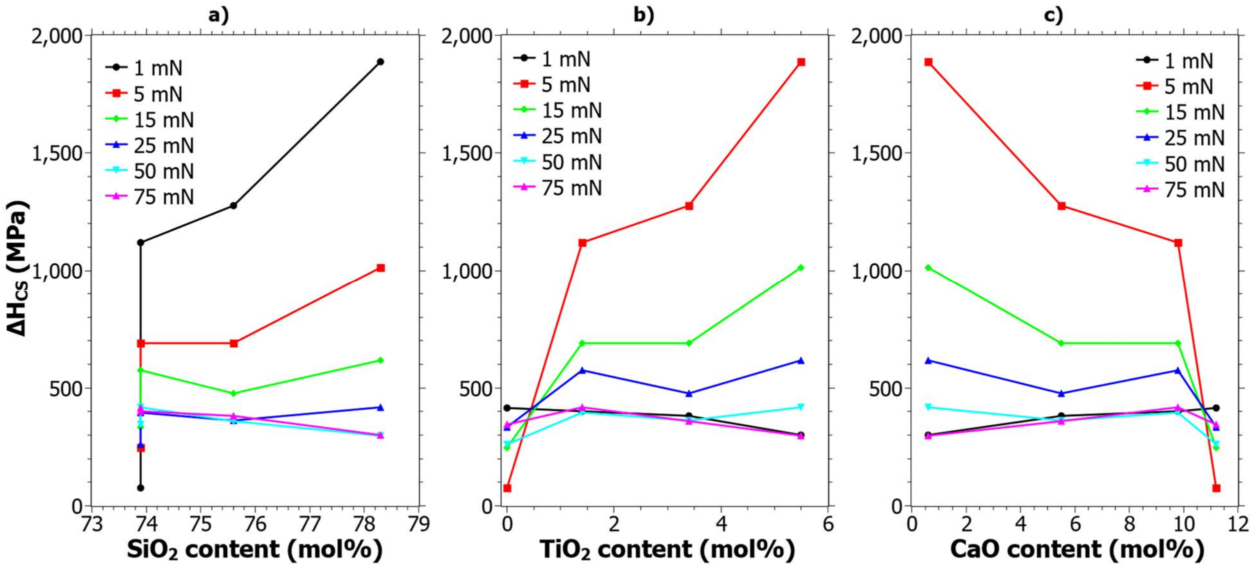

Figure S1. ΔH_{CS} of series 1 for different nanoindentation loads as a function of a) SiO_2, b) TiO_2 content and c) CaO content.

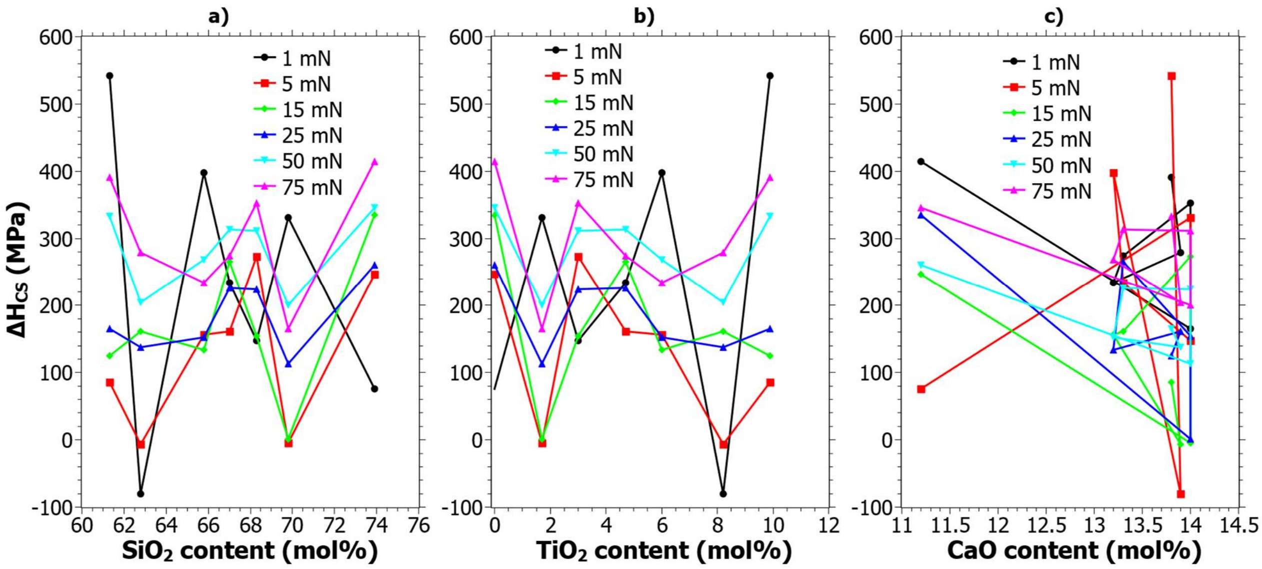

Figure S2. ΔH_{CS} of series 2 for different nanoindentation loads as a function of a) SiO_2, b) TiO_2 content and c) CaO content.

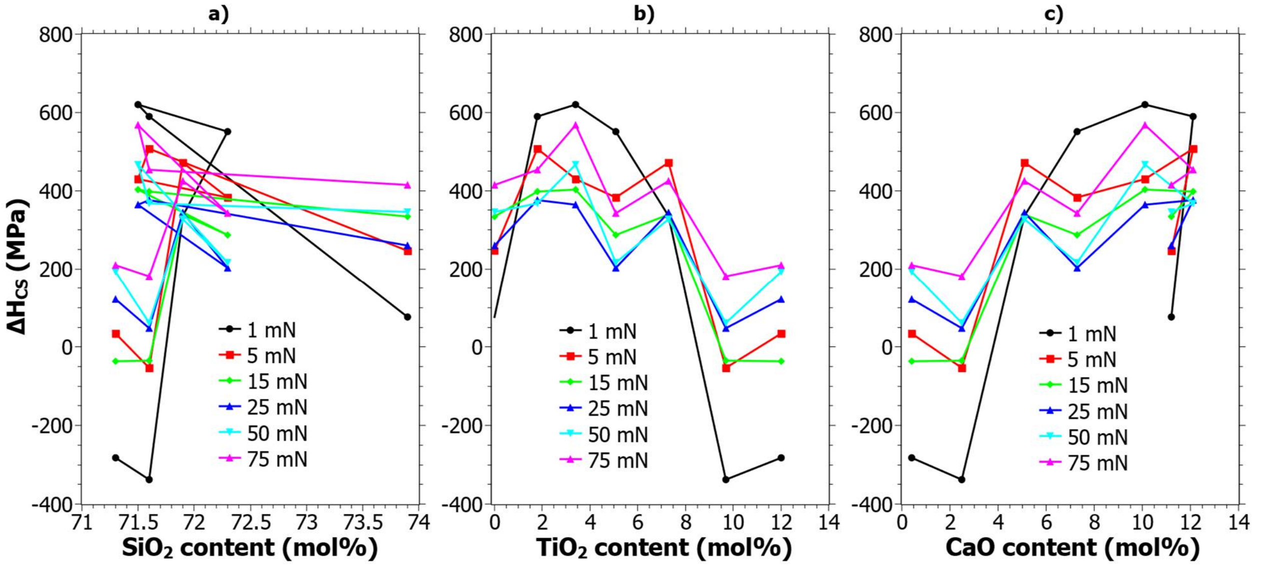

Figure S3. ΔH_{CS} of series 3 for different nanoindentation loads as a function of a) SiO_2, b) TiO_2 content and c) CaO content.

Table S1. The crack resistance (CR) results as well as fitting data xc (characteristic value) and m (Weibull modulus) for the Weibull fit.

Label	CR (N)	x_c	m
1.1	0.307	0.36	2.52
1.2	0.655	0.75	2.73
1.3	0.935	0.96	11.82
1.4	1.106	1.2	4.32
2.2	0.307	0.36	2.52
2.3	0.249	0.26	9.24
2.4	0.315	0.39	1.72
2.5	0.226	0.24	4.56
2.6	0.179	0.2	3.59
2.7	0.401	0.51	1.51
3.2	0.466	0.5	5.93
3.3	0.307	0.36	2.52
3.4	0.666	0.72	4.97
3.5	0.485	0.55	2.74
3.6	0.647	0.73	3.16
3.7	1.425	1.5	7.15
